# Targeted Therapy for Hypertension in Pregnancy: Hemodynamics as a Therapeutic Compass

**DOI:** 10.1016/j.jacadv.2025.102524

**Published:** 2026-01-17

**Authors:** Michael Ceulemans, Ruben Heremans, Chahinda Ghossein, Wilfried Gyselaers

**Affiliations:** a KU Leuven, Leuven, Belgium; b Research Foundation Flanders (FWO), Brussels, Belgium; c Ziekenhuis Oost Limburg Campus Maas en Kempen, Maaseik, Belgium; d Hartcentrum Hasselt, Hasselt, Belgium; e Cardiovascular Institute, Erasmus MC, Rotterdam, the Netherlands; f Hasselt University, Hasselt, Belgium

We commend Leonard et al^1^ for conducting a large target trial emulation in a real-world setting to assess the effectiveness and safety of labetalol and nifedipine for treating chronic hypertension during pregnancy. The study’s robust methodology and statistical rigor support the credibility of its findings based on available data. The authors correctly note that both medications are vasodilators, with labetalol also reducing cardiac output.

However, we would like to highlight an important clinical consideration. Previous research has shown that hypertensive disorders in pregnancy are not homogeneous; distinct hemodynamic subtypes exist, such as resistance-dominant and flow-dominant profiles.^2^ A recent meta-analysis on antihypertensive use in pregnancy did not account for these subtypes,^3^ perpetuating a one-size-fits-all approach and limiting the potential for targeted, personalized treatment. Yet, hemodynamic-guided therapy has led to faster and more effective blood pressure control compared to random treatment.^4^ In women with a history of preeclampsia, the recurrence rate was reduced by 45% with targeted pharmacotherapy compared with randomly selected antihypertensives.^5^

In their study, Leonard et al^1^ focused on pregnant individuals with chronic hypertension who initiated treatment before 20 weeks’ gestation. This cohort differs from the broader population at risk for hypertensive disorders in pregnancy, including patients without pre-existing hypertension but with elevated cardiovascular risk who may develop complications such as (pre)eclampsia or fetal growth restriction, which numerically far exceeds chronic hypertension in pregnancy. Additionally, we question whether differences in low-dose aspirin use between treatment groups may have influenced adverse outcomes.

In summary, we believe that without considering hemodynamic subtypes, studies on antihypertensive therapy in pregnancy ignore the critical dimension of individualized care. We agree with the authors that “selection of the medication should consider potential side effects, differences in regimen, and access,” but we would add that the patient’s hemodynamic profile should also be a key factor. We encourage the authors and other researchers to incorporate underlying pathophysiological mechanisms into study designs and data collection. In the meantime, it would also be valuable to understand how clinicians in the United States currently choose between antihypertensive agents during pregnancy and whether assessing a patient’s hemodynamic subtype is a feasible and implementable strategy in clinical practice.

## References

[1] Leonard S.A., Siadat S., Huybrechts K.F. (2025). Comparative effectiveness and safety of labetalol versus nifedipine for treatment of chronic hypertension during pregnancy. JACC Adv.

[2] Masini G., Foo L.F., Tay J. (2022). Preeclampsia has two phenotypes which require different treatment strategies. Am J Obstet Gynecol.

[3] Hup R.J., Damen J.A.A., Terstappen J. (2025). Oral antihypertensive treatment during pregnancy: a systematic review and network meta-analysis. Am J Obstet Gynecol.

[4] di Pasquo E., Giannubilo S.R., Valentini B. (2024). The “Preeclampsia and Hypertension Target Treatment” study: a multicenter prospective study to evaluate the effectiveness of the antihypertensive therapy based on maternal hemodynamic findings. Am J Obstet Gynecol MFM.

[5] Mulder E., Ghossein-Doha C., Gauffman E. (2021). Preventing recurrent preeclampsia by tailored treatment of nonphysiologic hemodynamic adjustments to pregnancy. Hypertension.

